# Identification of gene modules and hub genes associated with *Colletotrichum siamense* infection in mango using weighted gene co-expression network analysis

**DOI:** 10.1186/s12864-023-09811-6

**Published:** 2023-11-23

**Authors:** Zongling Liu, Zhengjie Zhu, Yuanhe Huang, Song Nong, Minli Jiang, Sangui Yi, Delong Xie, Hongliu Hu

**Affiliations:** 1 School of Basic Medical Sciences, Youjiang Medical University for Nationalities, Baise, 533000 China; 2 Guangxi Key Laboratory of Biology for Mango, Baise, 533000 China

**Keywords:** Weighted gene co-expression network analysis, Transcriptome, *Colletotrichum siamense*, Hub genes, Effectors, Mango

## Abstract

*Colletotrichum siamense* is a hemibiotrophic ascomycetous fungus responsible for mango anthracnose. The key genes involved in *C. siamense* infection remained largely unknown. In this study, we conducted weighted gene co-expression network analysis (WGCNA) of RNA-seq data to mine key genes involved in *Colletotrichum siamense*-mango interactions. Gene modules of Turquoise and Salmon, containing 1039 and 139 respectively, were associated with *C. siamense* infection, which were conducted for further analysis. GO enrichment analysis revealed that protein synthesis, organonitrogen compound biosynthetic and metabolic process, and endoplasmic reticulum-related genes were associated with *C. siamense* infection. A total of 568 proteins had homologs in the PHI database, 370 of which were related to virulence. The hub genes in each module were identified, which were annotated as O-methyltransferase (Salmon) and Clock-controlled protein 6 (Turquoise). A total of 24 proteins exhibited characteristics of SCRPs. By using transient expression in *Nicotiana benthamiana*, the SCRPs of XM_036637681.1 could inhibit programmed cell death (PCD) that induced by BAX (BCL-2-associated X protein), suggesting that it may play important roles in *C. siamense* infection. A mango-*C. siamense* co-expression network was constructed, and the mango gene of XM_044632979.1 (auxin-induced protein 15A-like) was positively associated with 5 SCRPs. These findings help to deepen the current understanding of necrotrophic stage in *C. siamense* infection.

**Supplementary Information:**

The online version contains supplementary material available at 10.1186/s12864-023-09811-6.

## Introduction

Mango (*Mangifera indica* L.)  is a kind of an important tropical fruits worldwide. Mango anthracnose, caused by *Colletotrichum* spp., is the most important disease of mango around the world, a disease responsible for a dramatic loss of crop yield. It has been reported that *Colletotrichum siamense* is one of the *Colletotrichum* species causing Mango anthracnose in Mexico, Thailand and China [1–3]. Mango anthracnose could attack leaves, twigs, flowering panicles, and fruits [2, 4]. Besides, the postharvest fruits infected with anthracnose caused greater economic losses. In the early stage of infection, the black spots would occur, following by enlargement and fusion of the spots. Eventually, it would cause the leaves and inflorescences to wither and the fruits to become rotting. Furthermore, mango anthracnose has latent infectivity, and the fruits, that seem healthy, would exhibit spots during post-harvest storage [5]. To control mango anthracnose, it is important to deepen the current understanding of the pathogenesis of the *Colletotrichum* spp..

Plant pathogen fungi would secret effectors to interfere the host immune system for infection [6, 7]. Small secreted cysteine-rich proteins (SCRPs) are a kind of known apoplastic effectors of pathogen fungi, whose cysteine residues could form disulphide bonds that enhance the stability in the apoplastic space [8, 9]. For instance, three SCRPs of *Verticillium dahliae* could cause cell death of *Nicotiana benthamiana* accompanied by a reactive oxygen species burst, callose deposition, and induction of defense genes [10]. Besides, the SCRPs of SCRE1 and SCRE2 secreted by *Ustilaginoidea virens* could inhibit programmed cell death (PCD) of *N. benthamiana* induced by BAX [11, 12]. The SCRPs played an important role during fungal infection. A lack of sufficient knowledge on SCRPs has prevent a deep understanding of the pathogenesis of *C. siamense* in mango.

Weighted gene co-expression network analysis (WGCNA) is an efficient method for mining modules and hub genes related to fungal infection [13, 14]. In the current study, we have conducted a WGCNA of RNA-seq data, aiming to identify gene modules and hub genes associated with *Colletotrichum siamense* infection in mango. The virulence-related genes were identified by PHI database BLASTp analysis. Additionally, we identified SCRPs from the genes in interested gene modules according to the characteristics of SCRPs [10]. Transient expression in *N. benthamiana* was used to screen the SCRPs that could interfere programmed cell death (PCD). A co-expression network was constructed to analyze the mango-*C. siamense* interactions. These findings will help to deepen the current understanding of the pathogenesis of *Colletotrichum siamense* in mango.

## Materials and methods

### RNA-seq data collection and processing

The raw RNA-seq data of *Colletotrichum siamense* infection in mango were achieved from NCBI (Bioproject: PRJNA872313), and the samples containing *Colletotrichum siamense* were chosen for further analysis. The fastq files were obtained by decompressing SRA files using software SRA toolkit v3.0.5. Then, the reads with low quality and adapts were removed using software fastp v0.17.0 [15]. The filtered reads of *C. siamense* hyphae and conidia samples were mapped to *C. siamense* genome (Genbank: GCF_013390195.1), and the filtered reads of *C. siamense* infection samples were mapped to both *C. siamense* genome and mango genome (Genbank: GCF_011075055.1) using software kallisto v0.46.0 [16]. The transcripts per kilobase million (TPM) values were calculated using software kallisto v0.46.0. The Linux command line code was deposited in Zenodo (https://doi.org/10.5281/zenodo.8424504). Differentially expressed genes (DEGs) were analyzed using R package of DESeq2 in TBtools v1.10 [17], and genes with *p*-value (adjusted) < 0.01 and |log2FC|> 2 were considered as DEGs. All genes were annotated using eggNOG v2.1.9 software [18].

### Weighted gene co-expression network analysis

The top 5000 genes according to median absolute deviation were used for following analysis. The R package of WGCNA [19] was used for weighted gene co-expression network analysis with a softpower value of 12 and a mergeCutHeight value of 0.25. The R script was deposited in Zenodo (https://doi.org/10.5281/zenodo.8424504). The genes were clustered into different gene modules according to different expression patterns. The *C. siamense* samples of conidia, hypha, and infecting mango for 3 d and 5 d status were considered as conidia, hypha, infect_3d, and infect_5 d traits, respectively. The module-trait correlation coefficient was calculated using the Pearson correlation analysis. The hub genes of significant modules were determined using the Cytohubba pluggin [20] in Cytoscape v3.9.1. To interpret the biological significance of genes in significant modules, Gene Ontology (GO) enrichment analysis was performed using TBtools v1.10.

### Prediction of candidate effectors

Genes in interested modules were selected for effector prediction. Firstly, the gene sequences were transformed into protein sequences, which then were conducted to BLASTp analysis using the Pathogen Host Interaction (PHI) database. Secondly, proteins with following characteristics [13] were considered as small secreted cysteine-rich proteins (SCRPs): (1) molecular mass is smaller than 400 amino acid (AA) residues, (2) cysteine content was greater than 3%, which was calculated using software DiANNA v1.1 (http://clavius.bc.edu/~clotelab/DiANNA/), (3) presence of an N-signal peptide, which was predicted using software SignalP v4.1 (https://services.healthtech.dtu.dk/service.php?SignalP-4.1), (4) absence of a transmembrane domain, which was predicted using software TMHMM v2.0 (https://services.healthtech.dtu.dk/service.php?TMHMM-2.0). Thirdly, the predicted SCRPs were conducted novel effectors prediction using software EffectorP v3.0 [21].

### *C. siamense*-mango gene co-expression network construction

The TPM gene expression levels of *C. siamense* predicted SCRPs genes and all mango genes were used for the gene co-expression network construction. The data were conducted Pearson pairwise correlation analysis across the selected samples using R packages of corrplot and hclust with |*R*|> 0.8 and *p*-value < 0.001, and the co-expression network of SCRPs genes with top 10 correlation coefficient values were visualized using software Cytoscape v3.5.1.

### Transient expression in *N. benthamiana*

A total of 10 cDNA sequences of predicted effectors were artificially synthesized, then they were cloned into the plasmid pCAMBIA3300-CaMV 35S in Beijing Tsingke Biotech Co., Ltd. The recombinant plasmids were transformed into competent cells of *Agrobacterium tumefaciens* strain GV3101 (AngYuBio) following the manufacturer’s instructions. Colonies with recombinant constructs were grown overnight in YEP medium (1% beef extract, 1% yeast extract, 0.5% NaCl [pH 7.0]) containing 50 μg/mL kanamycin and 20 μg/mL rifampicin. The cells were resuspended two times with infiltration buffer (10 mM MgCl_2_, 10 mM MES, and 150 µM acetosyringone). The cells with 35S::effectors were syringe-infiltrated alone into 4–5-week-old *N. benthamiana* leaves at OD600 = 0.6. Besides, the cells with 35S::effectors and 35S::BAX were syringe-infiltrated together with the volume ratio (1:1) at OD600 = 0.6. The cells with 35::GFP were considered as control. Leaf apoptosis was observed after 5 d of infiltration.

## Results

### *Analysis of RNA-*seq* data*

To reveal the transcriptome response of *C. siamense* infection, RNA-seq data of *C. siamense*-mango interactions were analyzed. In this dataset, a total of 24 samples belonging to *C. siamense*, including strains of GD10 and YN56, were selected for further analysis, 6 of which were status of conidia, 6 were status of hypha, 6 were status of 3 d infection, and 6 were status of 5 d infection. A total of 1746659580 clean reads were obtained, in which Q30 values were > 92.95%, and GC contents were > 43.27% (Table S1). The clean reads of samples of hypha and conidia were mapped to the *C. siamense* genome at a range of 83.5% to 87.2%, and samples of infection were 0.1% to 17.2% (Table S1). Additionally, the clean reads of samples of infection were mapped to the mango genome at a range of 62.9% to 88.2% (Table S1). These results suggested that the RNA-seq data were reliable for further analysis.

After 3 d and 5 d infection of mango, GD10-infection samples expressed 1254 and 1743 DEGs relative to GD10-hypha samples, and 1427 and 3427 DEGs relative to GD10-conidia samples, respectively, while YN56-infection samples expressed 1613 and 1737 DEGs relative to YN56-hypha samples, and 2854 and 2892 DEGs relative to YN56-conidia samples, respectively (Table 1). These results suggested that the transcriptome response of *C. siamense* infection was impressed.

**Table 1:** Statistics of differentially expressed genes

Group	Upregulated	Downregulated	All
GD10_hypha vs. GD10_infect3d	291	963	1254
GD10_hypha vs. GD10_infect5d	961	782	1743
GD10_conidia vs. GD10_infect3d	463	964	1427
GD10_conidia vs. GD10_infect5d	1516	1911	3427
YN56_hypha vs. YN56_infect3d	774	839	1613
YN56_hypha vs. YN56_infect5d	892	845	1737
YN56_conidia vs. YN56_infect3d	1158	1696	2854
YN56_conidia vs. YN56_infect5d	1406	1486	2892

### Identification of gene modules associated with C. siamense infection

**Fig. 1:**
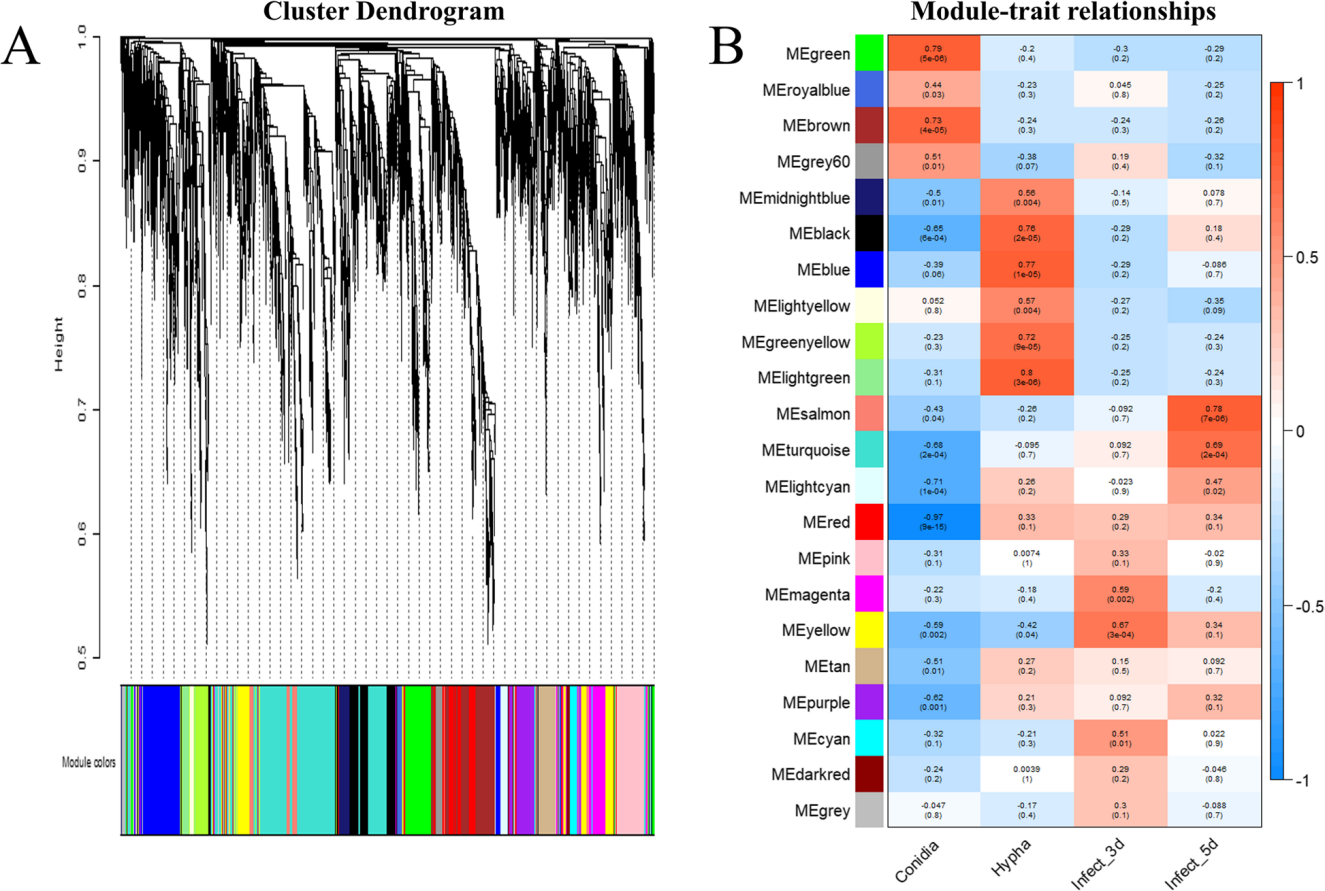
Identification of key gene modules associated with *C. siamense* infection. **A** Identification of gene modules by co-expression analysis. A total of 22 distinct gene modules were identified. **B** Correlation of gene modules and traits (Hypha, Conidia, Infect_3d, and Infect_5d). The correlation coefficient and adjusted *p*-value are shown in each cell

WGCNA was used to obtain gene modules associated with *C. siamense* infection in the mango. The expression profiles of top 5000 genes of median absolute deviation were used for co-expression module construction in WGCNA. According to the expression patterns, a total of 22 distinct gene modules were identified (Fig. 1A). To identify the gene modules related to *C. siamense* infection, the correlation coefficient between traits and gene modules were calculated. The results indicated that the Salmon and Turquoise modules were significantly and positively related to *C. siamense* infection trait of ‘Infect_5d’ (*r* = 0.78, *p* = 7e-06 for Salmon and *r* = 0.69, *p* = 2e-04 for Turquoise), while other gene modules were not (*p* > 0.01) (Fig. 1B). The Salmon and Turquoise modules contained 139 and 1039 genes, respectively, which were selected for subsequent analysis.

### Annotation of genes in interested modules

**Fig. 2:**
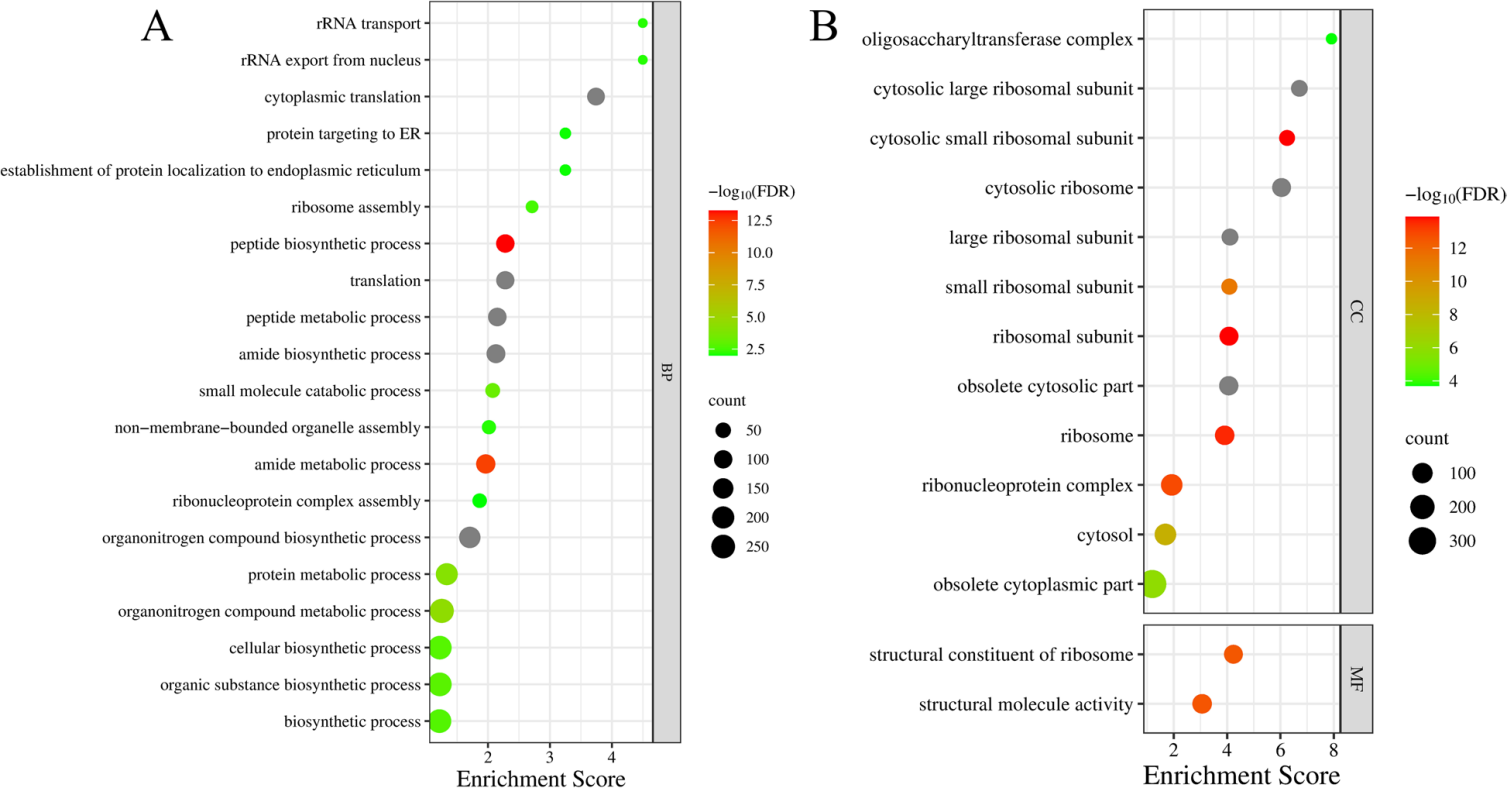
Enriched GO terms in Turquoise module. The columns and rows indicate GO terms and enrichment score. The round size indicates the number of hit genes. BP, CC, and MF represent biological process, cellular component, and molecular function. A total of 34 GO terms were significantly enriched (*FDR*-value < 0.01), and the details of enriched GO terms were shown in Table S2

GO enrichment analysis were used to explore the function of genes in Salmon and Turquoise modules. In the Salmon module, no GO term enrichment was identified. In the MEtuoquoise module, 34 GO terms were significantly enriched (*FDR* < 0.01), which were related to protein synthesis, ribosome, rRNA, organonitrogen compound biosynthetic and metabolic process, and endoplasmic reticulum (Fig. 2 and Table S2). The results suggested that a large amount of proteins were synthesis during *C. siamense* infection, which may be involved in hypha growth. Besides, some synthetized protein may be effectors secreted by endoplasmic reticulum, which play an important role in *C. siamense* colonization in mango.

### Hub genes in interested modules

**Fig. 3:**
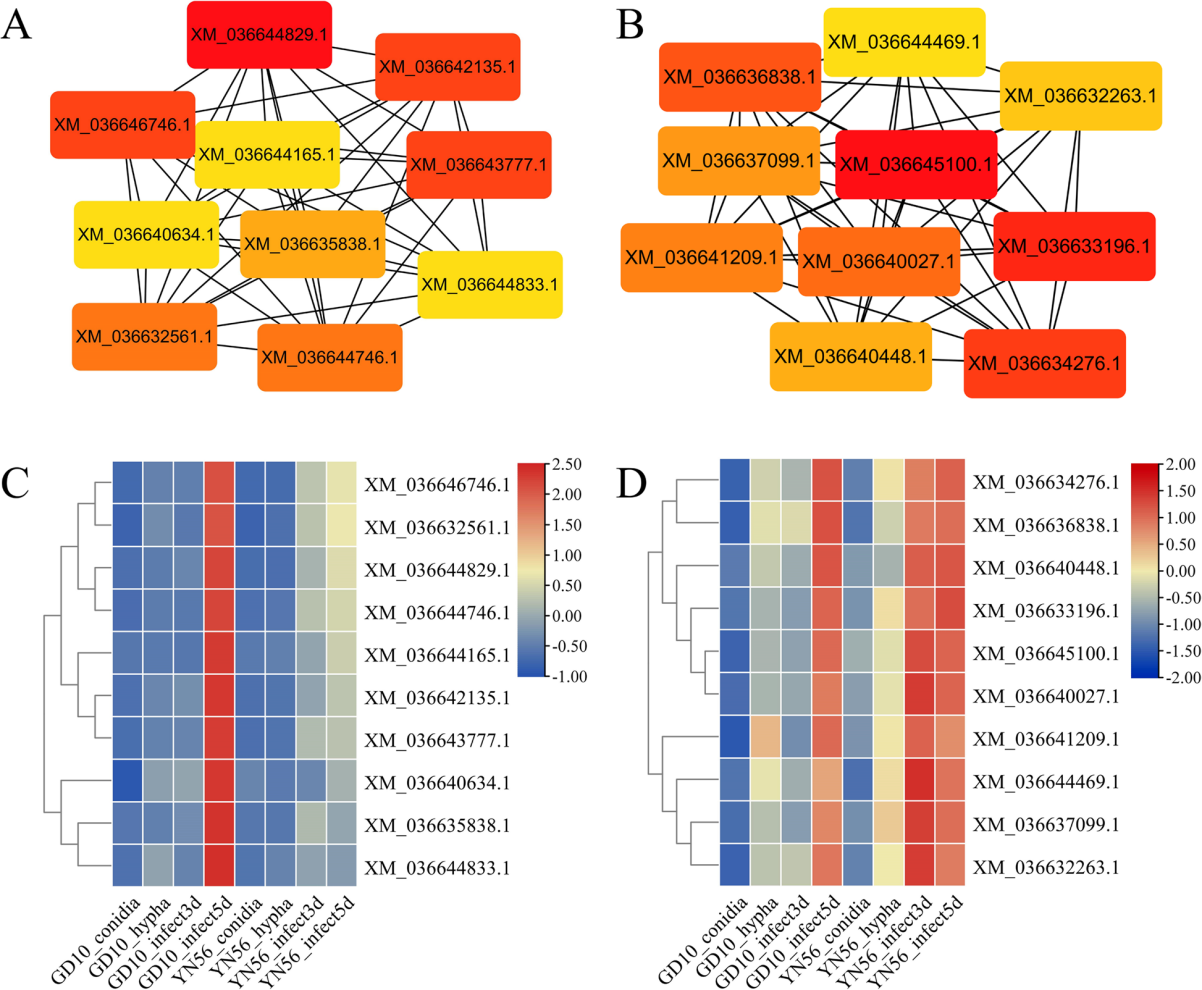
The top ten hub genes in the Salmon and Turquoise modules. Co-expression networks of top ten hub genes in the Salmon and Turquoise modules were shown in A and B, respectively. The details of hub genes were shown in Table S3. The cells are color-coded by degree value, which represents the number of connections in the co-expression network. Expression profiles of top ten hub genes in the Salmon and Turquoise modules were shown in C and D, respectively. The expression levels were standardized by rows

Hub genes were identified from Turquoise and Salmon gene modules. Gene co-expression networks of these modules were separately constructed, and the top ten hub genes in each module were identified based on the degree values by using the plugin Cytohubba. The top ten hub genes in each module were identified according to the degree value (Fig. 3A and B). In the Salmon module, the gene with highest degree value was XM_036644829.1, encoding O-methyltransferase (Table S3), which exhibited highest expression level with 5 d infection of GD10 (Fig. 3C). In the Turquoise module, the gene with highest degree value was XM_036645100.1, encoding Clock-controlled protein 6 (Table S3), and three of the hub genes were annotated as 60S ribosomal protein. The expression levels of these genes increased with a longer *C. siamense* infection time (Fig. 3D).

### Proteins in interested modules associated with virulence

**Fig. 4:**
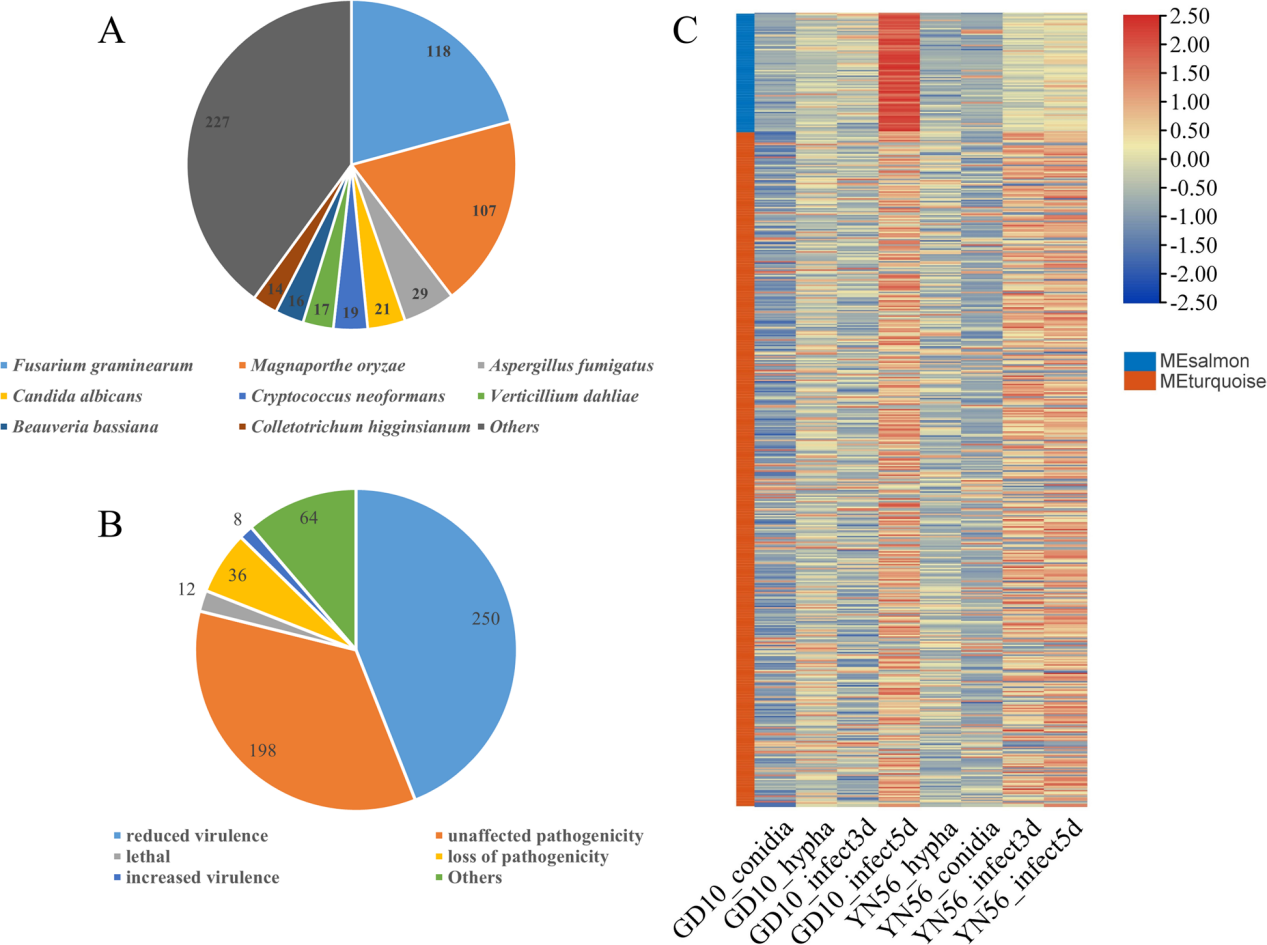
Homologous proteins identified in the Pathogen-Host Interaction (PHI) database. **A** Species distribution of homologous proteins in the PHI database. **B** Virulence distribution of homologous proteins in the PHI database. **C** Expression profiles of homologous proteins during *C. siamense* infection in mango. The expression levels were standardized by rows. The details of homologous proteins were shown in Table S4

To identify candidate proteins associated with virulence, proteins in the Salmon and Turquoise modules were BLASTp against the PHI database. A total of 568 proteins with significant homology were identified (Table S4), of which 227 and 118 were from *Aspergillus fumigatus* and *Fusarium graminearum* (Fig. 4A). According to the PHI database, by using gene deletion or silencing, 250 proteins exhibited reduced virulence, 198 proteins had no impact on pathogenicity, 36 proteins showed a loss of pathogenicity, and 8 proteins showed increased virulence (Fig. 4B). The homologs that identity value > 0.85 of XM_036634636.1, XM_036638449.1, XM_036644019.1, XM_036640956.1, XM_036638455.1, XM_036636947.1, and XM_036637134.1 showed a loss of pathogenicity, which were annotated as uncharacterized protein, plasma membrane ATPase, uncharacterized protein, superoxide-generating NADPH oxidase heavy chain subunit A, transcription factor steA, imidazoleglycerol-phosphate dehydratase, and uncharacterized protein (Table S4), respectively. The genes encoding these proteins were upregulated during *C. siamense* infection (Fig. 4C).

### Small secreted cysteine-rich proteins identified in interested modules

**Fig. 5:**
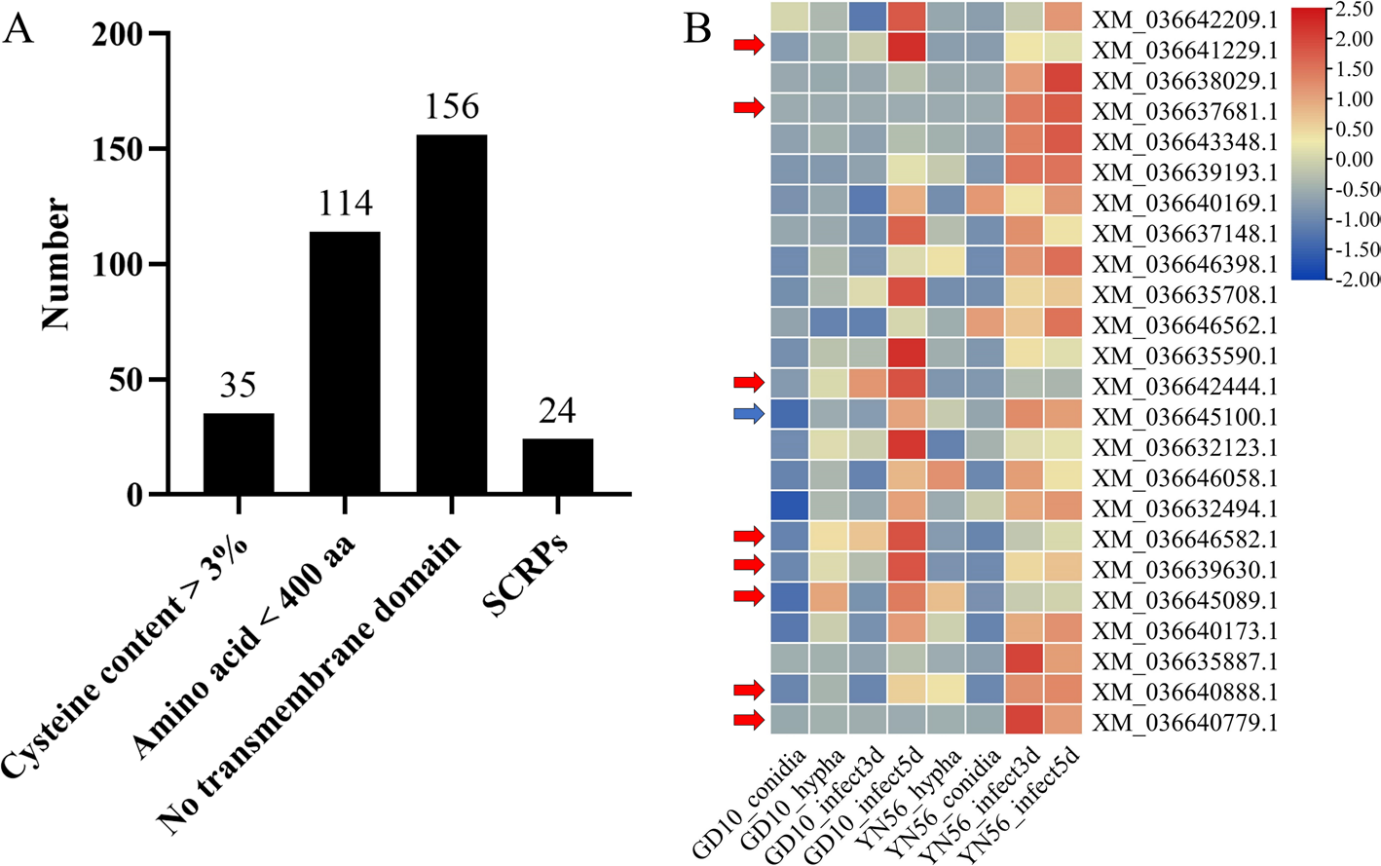
Small cysteine-rich proteins (SCRPs) in the Salmon and Turquoise modules. **A** In 193 proteins with N-signal peptide from these two modules, 35 had > 3% cysteine content, 114 had < 400 amino acids, 156 had no transmembrane domain. A total of 24 proteins exhibited all SCRPs characteristics. **B** Expression profiles of the predicted SCRPs genes. The expression levels were standardized by rows. The red arrows indicate homologous proteins identified in the PHI database. The blue arrow indicates hub genes identified by the Cytohubba plugin. The details of SCRPs were shown in Table S5

The small secreted cysteine-rich proteins (SCRPs) of *C. siamense* were predicted from 1178 proteins in the Salmon and Turquoise modules based on the characteristics of SCRPs. Among them, 193 proteins containing a N-signal peptide were conducted for further analysis. Of these, 35 proteins exhibited > 3% cysteine content, 156 proteins had no transmembrane domain, and 114 proteins had a molecular mass of < 400 aa (Fig. 5A and Table S5). Based on SCRPs characteristics as previously described, a total of 24 proteins were considered as SCRPs, 17 of which were annotated as uncharacterized proteins while the rest were annotated as CFEM domain protein (XM_036641229.1 and XM_036646582.1), Rodlet protein (XM_036642444.1), Clock controlled protein (XM_036645100.1), Acetylxylan esterase (XM_036640888.1), and LysM domain-containing protein (XM_036640779.1) (Table S5), of which 19 were predicted as novel effectors by using software EffectorP v3.0 (Table S5). The genes encoding these SCRPs were upregulated during *C. siamense* infection (Fig. 5B).

### Inhibition effect of XM_036637681.1 in PCD induced by BAX

**Fig. 6:**
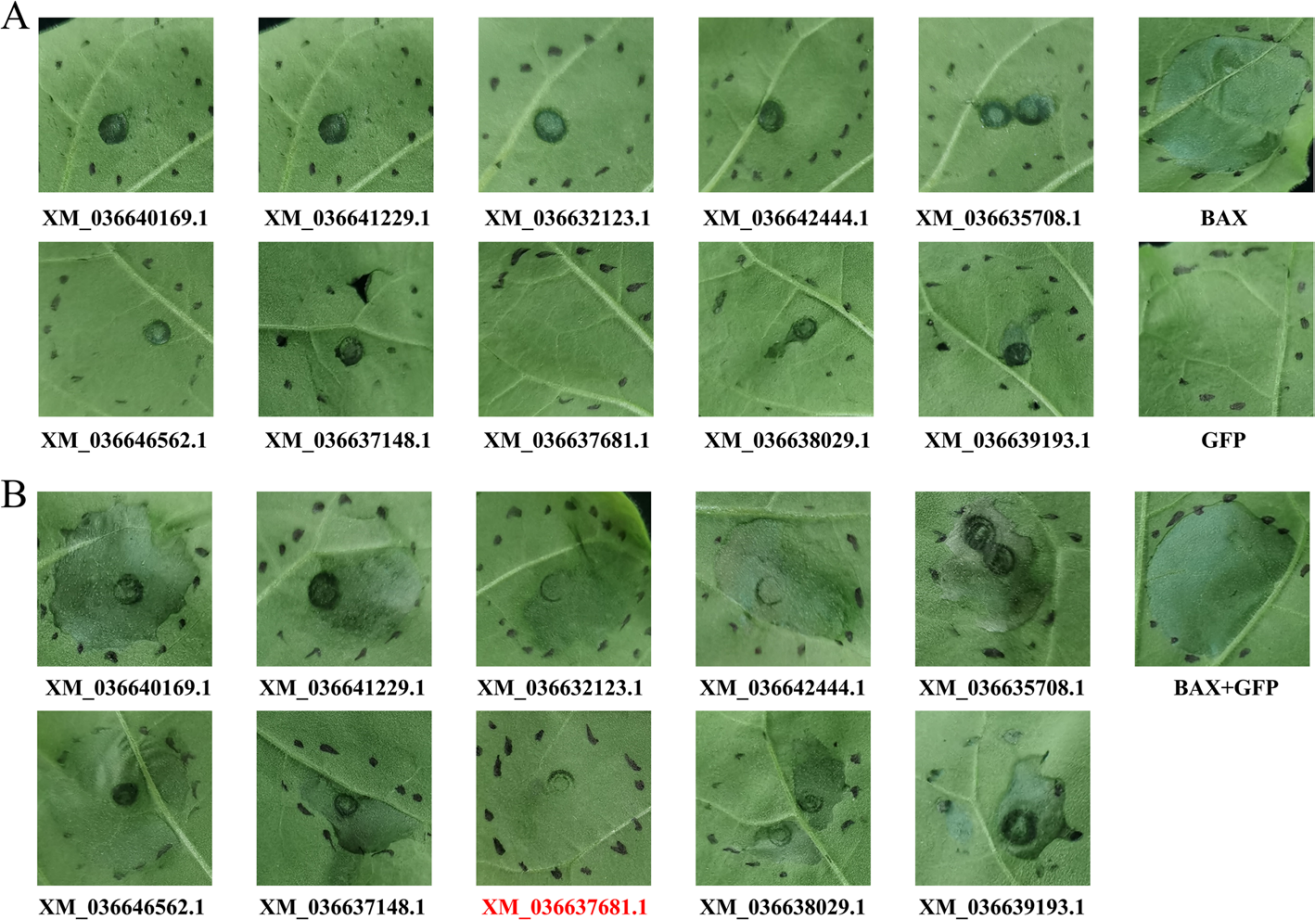
SCRPs of XM_036637681 could inhibit PCD induced by BAX. **A** Identification of PCD-inducing effectors. *Agrobacterium tumefaciens* harboring 35S::effector constructs infiltrating *Nicotiana benthamiana* leaves. *A. tumefaciens* harboring 35S::GFP and *A. tumefaciens* harboring 35S::BAX were used as negative and positive controls, respectively. **B** Identification of PCD-inhibiting effectors. *A. tumefaciens* containing the 35S::effector construct was mixed with *A. tumefaciens* containing the 35S::BAX construct and co-infiltrated into the leaves of *N. benthamiana*. *A. tumefaciens* harboring 35S::GFP mixed with *A. tumefaciens* harboring 35S::BAX were used as the positive control. The photographs were taken 5 d after infiltration. The assays were performed in triplicates

To analyze the potential function of the predicted SCRPs, a total of 10 SCRPs (without signal peptide) were subjected to PCD screening on *N. benthamiana* using a transient expression system. All of them could not induce PCD on *N. benthamiana* (Fig. 6A). The SCRPs of XM_036637681.1 could inhibit PCD that induced by BAX (Fig. 6B). XM_036637681.1 was annotated as uncharacterized protein with 7.69% cysteine percentage and predicted as apoplastic effector by using software Effector P v3.0 (Table S5), and the most significantly positive co-expressed mango gene was XM_044632979.1 with correlation coefficient value of 0.9516, annotated as auxin-induced protein 15A-like (Table S6). These results suggested that XM_036637681.1 may play an important role in *C. siamense* infection in mango.

### Co-expression network of SCRPs and mango genes

**Fig. 7:**
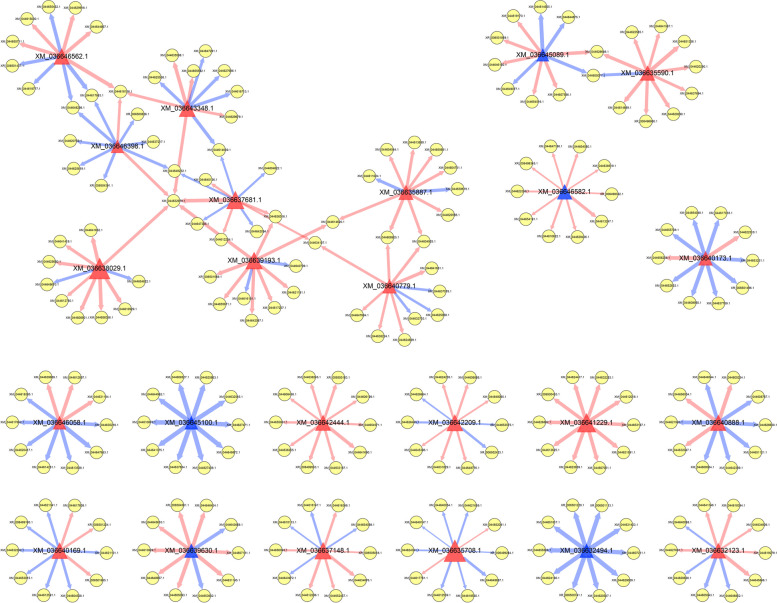
Co-expression network of SCRPs genes and mango genes. The co-expression of top 10 correlation coefficient values of each SCRPs were visualized using software Cytoscape v3.5.1. The red and blue triangles indicate effector and non-effector, respectively, which were predicted by software EffectorP v3.0. The yellow rounds indicate mango genes. The red and blue lines indicate positively and negatively regulation. Thicker lines indicate higher correlation coefficients. The details of these genes were shown in Table S6

To explore co-expression network of SCRPs and mango genes, the correlation was analyzed using the Pearson method with absolute correlation coefficients > 0.8 and *p* value < 0.001, and the co-expression of top 10 correlation coefficient values of each SCRPs were visualized using Cytoscape v2.4.1. All 24 SCRPs genes exhibited both positive and negative co-expression of mango genes, 13 of which were co-expressed with unique mango genes while others were not (Fig. 7). In the co-expression network, there were 11, 1, and 1 mango genes co-expressed with 2, 3, and 5 SCRPs genes, respectively (Fig. 7), and other mango genes were only co-expressed with 1 SCRPs gene. Notably, Mango gene of XM_044618156.1, annotated as uncharacterized protein, were positively co-expressed with SCRPs genes of XM_036646562.1, XM_036646398.1, and XM_036643348.1. Besides, mango gene of XM_044632979.1, annotated as auxin-induced protein 15A-like, were positively co-expressed with SCRPs genes of XM_036643348.1, XM_036646398.1, XM_036637681.1, XM_036638029.1, and XM_036639193.1. These results suggested that mango genes of XM_044618156.1 and XM_044632979.1 may play an important role in *C. siamense*-mango interactions.

## Discussion

*C. siamense* is a hemibiotrophic ascomycetous fungus, whose infection could be devided into primary biotrophic stage and secondary necrotrophic stage. According to sample descriptions of GCF_013390195.1, hyphae can be seen on the spots when sampling after 5 d inoculation, which could tell that it was in the secondary necrotrophic stage [22]. Therefore, we focused on transcriptome analysis of the secondary necrotrophic stage in the current study. WGCNA is a powerful tool to reveal the correlation between gene expression levels and phenotypic traits [14, 23]. Two gene modules (Salmon and Turquoise) significantly associated with *C. siamense* 5 d infection in mango (*p* < 0.001) were identified. Of particular interest were genes in Turquoise module that were enriched in protein synthesis, organonitrogen compound biosynthetic and metabolic process, and endoplasmic reticulum-related GO terms. As for protein synthesis-related GO terms, the necrotrophic hyphae grow rapidly leading to necrotic lesions, and numerous conidia would be produced in the secondary necrotrophic stage [24], which needs massive protein synthesis. Additionally, the protein synthesis-related genes may be involved in producing effectors and plant cell wall degradation enzyme, which were associated with virulence. As for endoplasmic reticulum-related GO terms, *Colletotrichum* spp. would secret plant cell wall degradation enzymes [25, 26] and effectors [27, 28] for colonization, and endoplasmic reticulum is the main organelle involved in the secretion. As for organonitrogen compound biosynthetic and metabolic process-related GO terms, it has been reported that nitrogen-containing metabolites produced by *Colletotrichum* spp. play an important role in fungi-plant interaction [29], such as Ferricrocin [30] and Melanin [31]. Thus, these studies implied that genes in these enriched GO terms play a critical role in *C. siamense* infection in mango.

Hub genes in each module were mined by using Cytohubba plugin. In the Salmon module, the hub gene (XM_036644829.1), with the highest degree value, was annotated as O-methyltransferase that is involved in methylation modification of oxygen atoms in secondary metabolites, which improved the stability for better biological activity [32]. During *Colletotrichum* spp. infection, it would secret some secondary metabolites for plant cell damage [29], and the O-methyltransferase may play an important role in methylation modification of these secondary metabolites. In the Turquoise module, the top hub gene (XM_036645100.1) was annotated as Clock-controlled protein 6, belonging to circadian clock proteins, that provided information of daily environment changes and controlled primary metabolisms [33, 34]. Interestingly, this protein exhibited characteristics of SCRPs, including N-signal peptide, implying that it may be secreted to host, and interfered the host primary metabolisms. Functional characterization of these two genes will be conducted in a future study.

The PHI database records pathogen proteins that are functionally characterized in pathogen-plant interactions [35]. Through a BLASTp analysis, 568 of 1178 proteins in the Salmon and Turquoise modules had homologous proteins in the PHI database, suggesting that there is a reliable association between virulence and these two gene modules, of which 36 proteins showed a loss of pathogenicity that may also play essential roles in *C. siamense* virulence. Interestingly, 8 proteins identified in the PHI database exhibited characteristics of SCRPs, suggesting that there is a reliable association between virulence and SCRPs.

High content of cysteine formed disulfide bond in the SCRPs, which kept it stable in the host environment [36], and SCRPs are a kind of effector that interferes with host immune system [10]. In the current study, 24 proteins had SCRPs characteristics, of which 18 proteins were annotated as uncharacterized protein, and they deserved further functional analysis. Two proteins (XM_036641229.1 and XM_036646582.1) were annotated as CFEM domain protein, which were effectors in pathogenic fungi [37]. Notably, the SCRPs of XM_036645100.1 belongs to hub genes, indicating that it played an important role in *C. siamense* infection.

Transient expression in *N. benthamiana* is an effective method to identify effectors [10]. In this study, by using transient expression in *N. benthamiana*, the SCRPs of XM_036637681.1, annotated as uncharacterized protein, could inhibit PCD that induced by BAX. Normally, plants form a ‘wall’ to prevent fungi hyphae expansion by PCD, while effector secreted by fungi would inhibit the PCD process for fungi colonization [7]. The SCRPs of XM_036637681.1 could inhibit PCD process in mango during *C. siamense* infection. The function of XM_036637681.1 will be further determined by gene deletion.

Co-expression network is a useful tool to study plant-fungi interaction. An interaction between a smut effector gene *SsPele1* and a sugarcane gene *ScPEPR1* was identified based on co-expression network [14]. A co-expression network between SCRPs genes and mango genes was constructed based on Pearson analysis. Notably, mango gene of XM_044632979.1, annotated as auxin-induced protein 15A-like, was positively associated with 5 SCRPs genes. Auxin is involved in plant-fungi interaction [38], and XM_044632979.1 is a part of auxin signaling, which may be regulated by SCRPs to increase host susceptibility. The relationship between XM_044632979.1 and 5 SCRPs deserves further study.

**Fig. 8:**
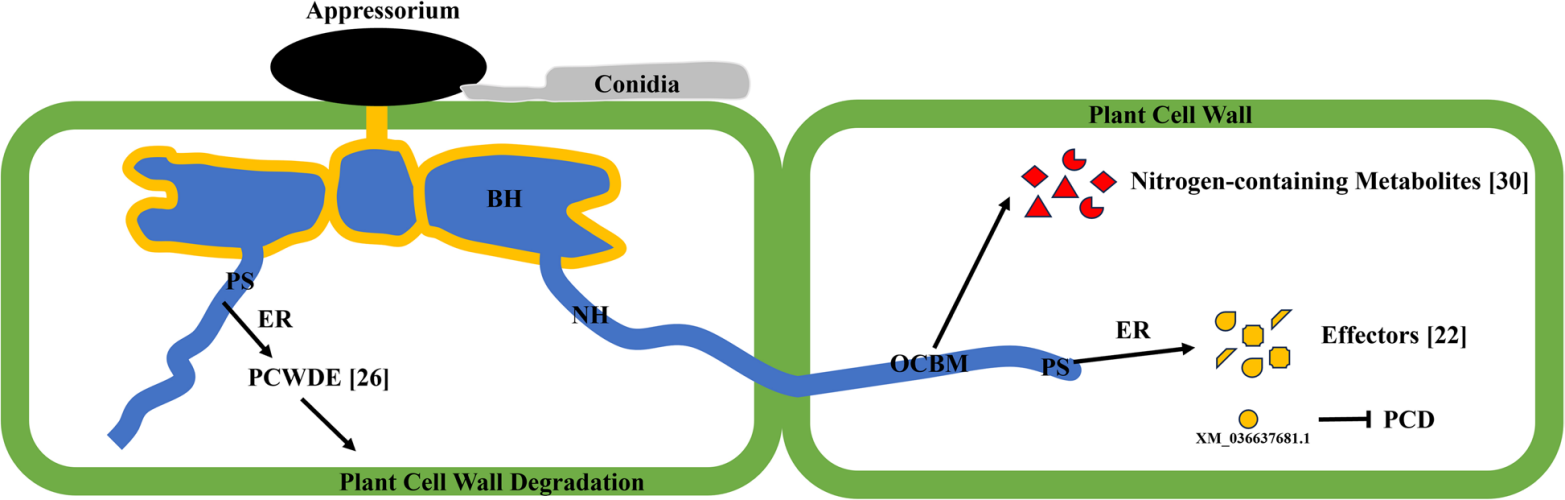
A postulated model of *C. siamense* infection in necrotrophic stage. The model was postulated based on current findings. BH indicates biotrophic hypha. NH indicates necrotrophic hypha. PS indicates protein-synthesis-related proteins. ER indicates endoplasmic reticulum-related proteins. PCWDE indicates plant cell wall degradation enzymes. OCBM indicates organonitrogen compound biosynthetic and metabolic process-related proteins. PCD indicates programmed cell death

Current findings let us to postulate a *C. siamense* infection model of necrotrophic stage (Fig. 8). Protein synthesis-related proteins would participate in plant cell wall degradation enzymes and effectors synthesis, and then endoplasmic reticulum-related proteins would assist in their transportations. PCWDEs were involved in plant cell degradation for colonization. Effectors interfered host immune system, XM_036637681.1 of which could inhibit programmed cell death (PCD) process. Furthermore, organonitrogen compound biosynthetic and metabolic process-related proteins were responsible in synthesis of nitrogen-containing metabolites, some of which would be toxic to plants [29].

In conclusion, two gene modules (Turquoise and Salmon), containing a total of 1178 genes, significantly associated with *C. siamense* infection of necrotrophic stage in mango were identified using WGCNA. GO enrichment analysis of genes in these modules revealed that protein synthesis, organonitrogen compound biosynthetic and metabolic process, and endoplasmic reticulum-related genes were implicated in *C. siamense* infection. The hub genes of XM_036644829.1 (O-methyltransferase, Salmon) and XM_036645100.1 (Clock-controlled protein 6, Turquoise) were identified using the Cytohubba plugin. Of 1178 proteins, 568 proteins were identified in PHI database, and 24 proteins exhibited SCRPs characteristics. The SCRPs of XM_036637681.1 could inhibit PCD that induced by BAX. Besides, the mango gene of XM_044632979.1 was positively associated with 5 SCRPs based on co-expression network. These findings help to deepen the current understanding of necrotrophic stage in *C. siamense* infection.

### Supplementary Information

**Additional file 1.**

## Data Availability

The datasets supporting the conclusions of this article are available in the National Center for Biotechnology Information repository, https://www.ncbi.nlm.nih.gov/bioproject/PRJNA872313.

## References

[1] Mo J, Zhao G, Li Q, Solangi GS, Tang L, Guo T, Huang S, Hsiang T (2018). Identification and characterization of *colletotrichum* species associated with mango anthracnose in Guangxi. China Plant Dis.

[2] Tovar-Pedraza JM, Mora-Aguilera JA, Nava-Díaz C, Lima NB, Michereff SJ, Sandoval-Islas JS, Câmara MPS, Téliz-Ortiz D, Leyva-Mir SG (2019). Distribution and pathogenicity of *colletotrichum* species associated with mango anthracnose in Mexico. Plant Dis.

[3] Rattanakreetakul C, Keawmanee P, Bincader S, Mongkolporn O, Phuntumart V, Chiba S, Pongpisutta R (2023). Two newly identified *colletotrichum* species associated with mango anthracnose in Central Thailand. Plants (Basel).

[4] Arauz LF (2000). Mango anthracnose: economic impact and current options for integrated managaement. Plant Dis.

[5] Cheng YJ, Wu YJ, Lee FW, Ou LY, Chen CN, Chu YY, Kuan YC (2022). Impact of storage condition on chemical composition and antifungal activity of pomelo extract against *colletotrichum gloeosporioides* and anthracnose in post-harvest mango. Plants (Basel).

[6] De Wit PJ, Mehrabi R, Van den Burg HA, Stergiopoulos I (2009). Fungal effector proteins: past, present and future. Mol Plant Pathol.

[7] Lo PL, Lanver D, Schweizer G, Tanaka S, Liang L, Tollot M, Zuccaro A, Reissmann S, Kahmann R (2015). Fungal effectors and plant susceptibility. Annu Rev Plant Biol.

[8] Saunders DG, Win J, Cano LM, Szabo LJ, Kamoun S, Raffaele S (2012). Using hierarchical clustering of secreted protein families to classify and rank candidate effectors of rust fungi. PLoS ONE.

[9] Cheng Y, Wu K, Yao J, Li S, Wang X, Huang L, Kang Z (2017). PSTha5a23, a candidate effector from the obligate biotrophic pathogen *Puccinia striiformis* f. sp. tritici, is involved in plant defense suppression and rust pathogenicity. Environ Microbiol.

[10] Wang D, Tian L, Zhang DD, Song J, Song SS, Yin CM, Zhou L, Liu Y, Wang BL, Kong ZQ (2020). Functional analyses of small secreted cysteine-rich proteins identified candidate effectors in *Verticillium dahliae*. Mol Plant Pathol.

[11] Fang A, Gao H, Zhang N, Zheng X, Qiu S, Li Y, Zhou S, Cui F, Sun W (2019). A novel effector gene SCRE2 contributes to full virulence of *Ustilaginoidea virens* to rice. Front Microbiol.

[12] Zhang N, Yang J, Fang A, Wang J, Li D, Li Y, Wang S, Cui F, Yu J, Liu Y (2020). The essential effector SCRE1 in *Ustilaginoidea virens* suppresses rice immunity via a small peptide region. Mol Plant Pathol.

[13] Liu Z, Li X, Li J, Zhao H, Deng X, Su Y, Li R, Chen B (2022). Identification of gene modules and hub genes associated with *Sporisorium scitamineum* infection using weighted gene co-expression network analysis. J Fungi (Basel).

[14] Ling H, Fu X, Huang N, Zhong Z, Su W, Lin W, Cui H, Que Y (2022). A sugarcane smut fungus effector simulates the host endogenous elicitor peptide to suppress plant immunity. New Phytol.

[15] Chen S, Zhou Y, Chen Y, Gu J (2018). fastp: an ultra-fast all-in-one FASTQ preprocessor. Bioinformatics.

[16] Bray NL, Pimentel H, Melsted P, Pachter L (2016). Near-optimal probabilistic RNA-seq quantification. Nat Biotechnol.

[17] Love MI, Huber W, Anders S (2014). Moderated estimation of fold change and dispersion for RNA-seq data with DESeq2. Genome Biol.

[18] Huerta-Cepas J, Szklarczyk D, Heller D, Hernández-Plaza A, Forslund SK, Cook H, Mende DR, Letunic I, Rattei T, Jensen LJ (2019). eggNOG 5.0: a hierarchical, functionally and phylogenetically annotated orthology resource based on 5090 organisms and 2502 viruses. Nucleic Acids Res.

[19] Langfelder P, Horvath S (2008). WGCNA: an R package for weighted correlation network analysis. BMC Bioinformatics.

[20] Ma H, He Z, Chen J, Zhang X, Song P (2021). Identifying of biomarkers associated with gastric cancer based on 11 topological analysis methods of CytoHubba. Sci Rep.

[21] Sperschneider J, Dodds PN (2022). EffectorP 3.0: prediction of apoplastic and cytoplasmic effectors in fungi and oomycetes. Mol Plant Microbe Interact.

[22] Yan Y, Yuan Q, Tang J, Huang J, Hsiang T, Wei Y, Zheng L (2018). *Colletotrichum higginsianum* as a model for understanding host-pathogen interactions: a review. Int J Mol Sci.

[23] Chen Q, Zhang R, Li D, Wang F (2021). Transcriptomic and coexpression network analyses revealed pine chalcone synthase genes associated with pine wood nematode infection. Int J Mol Sci.

[24] Münch S, Lingner U, Floss DS, Ludwig N, Sauer N, Deising HB (2008). The hemibiotrophic lifestyle of *Colletotrichum* species. J Plant Physiol.

[25] Wattad C, Kobiler D, Dinoor A, Prusky D (1997). Pectate lyase of *Colletotrichum gloeosporioides* attacking avocado fruits: cDNA cloning and involvement in pathogenicity. Physiol Mol Plant P.

[26] Kramer-Haimovich H, Servi E, Katan T, Rollins J, Okon Y, Prusky D (2006). Effect of ammonia production by *Colletotrichum gloeosporioides* on pelB activation, pectate lyase secretion, and fruit pathogenicity. Appl Environ Microbiol.

[27] de Jonge R, Thomma BP (2009). Fungal LysM effectors: extinguishers of host immunity?. Trends Microbiol.

[28] Tsushima A, Narusaka M, Gan P, Kumakura N, Hiroyama R, Kato N, Takahashi S, Takano Y, Narusaka Y, Shirasu K (2021). The conserved *colletotrichum* spp. effector candidate CEC3 induces nuclear expansion and cell death in plants. Front Microbiol.

[29] Kim JW, Shim SH (2019). The fungus *Colletotrichum* as a source for bioactive secondary metabolites. Arch Pharm Res.

[30] Ohra J, Morita K, Tsujino Y, Tazaki H, Fujimori T, Goering M, Evans S, Zorner P (1995). Production of the phytotoxic metabolite, ferricrocin, by the fungus *Colletotrichum gloeosporioides*. Biosci Biotechnol Biochem.

[31] Tsuji G, Sugahara T, Fujii I, Mori Y, Ebizuka Y, Shiraishi T, Kubo Y (2003). Evidence for involvement of two naphthol reductases in the first reduction step of melanin biosynthesis pathway of *Colletotrichum lagenarium*. Mycol Res.

[32] Cho JH, Park Y, Ahn JH, Lim Y, Rhee S (2008). Structural and functional insights into O-methyltransferase from *Bacillus cereus*. J Mol Biol.

[33] Mazzoccoli G, Pazienza V, Vinciguerra M (2012). Clock genes and clock-controlled genes in the regulation of metabolic rhythms. Chronobiol Int.

[34] Kim JA, Kim HS, Choi SH, Jang JY, Jeong MJ, Lee SI (2017). The importance of the circadian clock in regulating plant metabolism. Int J Mol Sci.

[35] Urban M, Cuzick A, Seager J, Wood V, Rutherford K, Venkatesh SY, De Silva N, Martinez MC, Pedro H, Yates AD (2020). PHI-base: the pathogen-host interactions database. Nucleic Acids Res.

[36] Lyu X, Shen C, Fu Y, Xie J, Jiang D, Li G, Cheng J (2016). A Small secreted virulence-related protein is essential for the necrotrophic interactions of *Sclerotinia sclerotiorum* with its host plants. PLoS Pathog.

[37] Zhang ZN, Wu QY, Zhang GZ, Zhu YY, Murphy RW, Liu Z, Zou CG (2015). Systematic analyses reveal uniqueness and origin of the CFEM domain in fungi. Sci Rep.

[38] Ludwig-Müller J (2015). Bacteria and fungi controlling plant growth by manipulating auxin: balance between development and defense. J Plant Physiol.

